# Liver transplantation and COVID-19: a case report and cross comparison between two identical twins with COVID-19

**DOI:** 10.1186/s12893-020-00837-1

**Published:** 2020-08-08

**Authors:** Hamed Nikoupour, Peyman Arasteh, Siavash Gholami, Saman Nikeghbalian

**Affiliations:** Shiraz Transplant Center, Abu Ali Sina Hospital, Shiraz University of Medical Sciences, Shiraz, Iran

**Keywords:** COVID-19, Transplantation, Liver, Prognosis

## Abstract

**Background:**

To this date little information exists on the effects, clinical course and outcome of the COVID-19 among patients undergoing transplantation.

**Case presentation:**

A 35 year old male referred with loss of sense of smell and taste after having close contact with his brother who was diagnosed with COVID-19 five days prior to his symptoms. The patient had undergone liver transplantation 3 years prior to his referral due to primary sclerosing cholangitis in association with ulcerative colitis and was using immunosuppressive medications. The patient referred to a local physician with mild symptoms of fatigue, cough, myalgia, dizziness, and nausea/vomiting with a fear of contracting the disease. Except for a CRP of 32 his other blood tests were normal. After 3 days of hospital admission the patient was discharged with a good condition. His brother had developed fever, chills, headache, mild dyspnea and an objective loss of sense of smell and taste and was sent home and advised to self-quarantine. Both patients had CT scans in favor of COVID-19.

**Conclusion:**

Our patient who had liver transplantation and COVID-19 did not present more severe symptoms compared to his counterpart without liver transplantation and did not need to be hospitalized or be given antiviral drugs for COVID-19.

## Background

Recently the world has been facing a new pandemic from a disease known as the coronavirus disease 2019 or COVID-19 caused by a novel corona virus which started from Wuhan, China [1]. The COVID-19 infection has rapidly spread to include most countries in the world [2].

Among the most sensitive groups to infections are those who have undergone transplantations [3]. To this date little information exists on the effects, clinical course and outcome of the COVID-19 among patients undergoing transplantation.

In here we report two cases of COVID-19 infections among two identical twin brothers one of whom previously had a liver transplantation.

## Case presentation

### Case number 1

A 35 year old male developed an objective loss of sense of smell and taste after having close contact with his brother who was diagnosed with COVID-19 five days prior to his symptoms. The patient had undergone liver transplantation 3 years prior to his referral due to primary sclerosing cholangitis in association with ulcerative colitis and was using immunosuppressive medications including 12.5 mg of prednisolone, 1440 mg of mycophenolic acid, and 4 mg of tacrolimus on a daily bases. He was also using other medications including 1 mg of folic acid, 1000 mg of mesalazine and 600 mg of ursobil. He had a B+ blood group. Previously the patient underwent an uneventful whole organ liver transplantation with a Model for End-Stage Liver Disease (MELD) score of 16 and a Child-Pugh score of 6 (class A).

He worked in an office with 29 other colleagues alongside his brother.

**Fig. 1:**
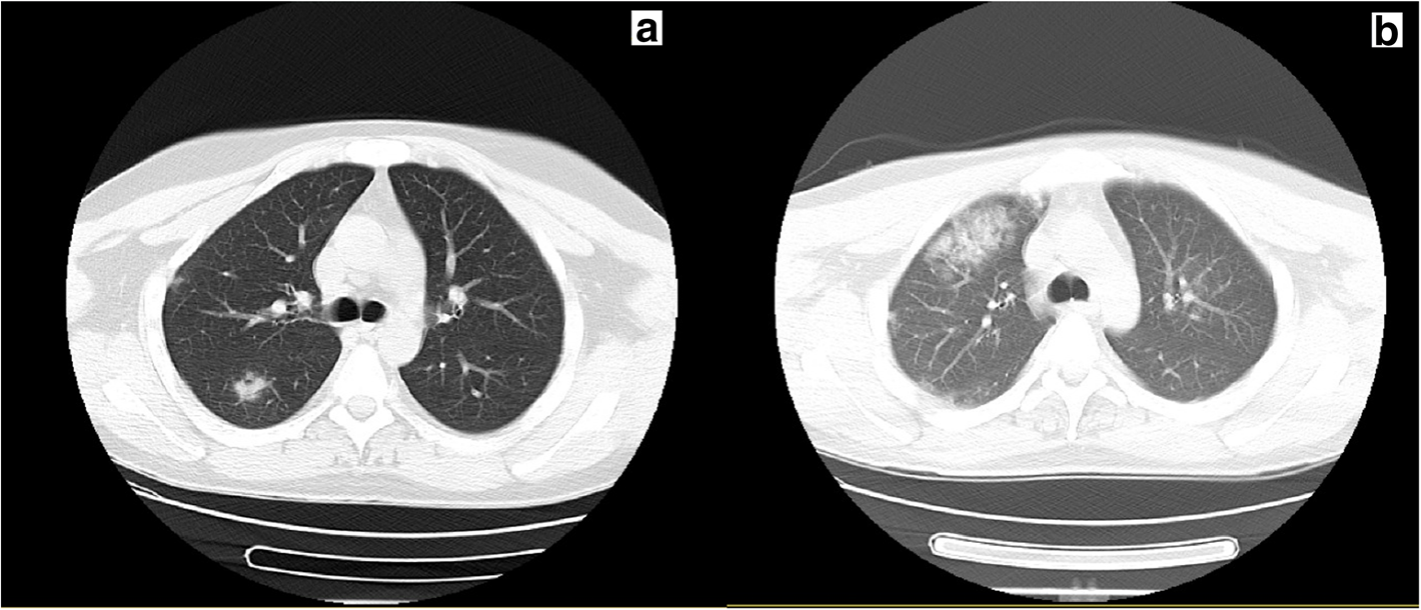
CT scan of the chest of the two identical twins with COVID-19. Figure “**a**” shows patient number 1 who previously had a liver transplantation and figure “**b**” shows patient number 2 who had no underlying disease. CT scan of patient “b” shows more severe pulmonary involvement by COVID-19

The patient referred to a local physician with mild symptoms of fatigue, cough, myalgia, dizziness, and nausea/vomiting with a fear of contracting the disease. During his visit a complete blood cell count, C-reactive protein (CRP) and a computed tomography (CT) scan of his chest was requested. He had a CRP of 32 mg/l and a CT scan which was in favor of COVID-19 (Fig. 1). The patient was then admitted to a hospital considering his immunosuppressed state.

During hospital admission the patient had a low grade fever of 38 degrees centigrade and a 98% oxygen saturation.

Patients’ lab data during hospital admission was as follows: white blood cell count (WBC): 6900unit/ml, neutrophil count: 3746unit/ml, lymphocyte count: 2635unit/ml, monocyte count: 517unit/ml, hemoglobin: 11.2 g/dl, platelet count: 149000, prothrombin time (PT): 13 s, international normalized ratio (INR): 1, partial thromboplastin time (PTT): 31 s, CRP: negative, blood urea nitrogen (BUN): 13 mg/dl, creatinine: 0.7 mg/dl, liver function tests included an aspartate transaminase (AST): 32 IU/L, alanine transaminase (ALT): 12 IU/L, alkaline phosphatase (ALK): 99 IU/L, total bilirubin: 2.4 mg/dl and direct bilirubin: 0.4 mg/dl, electrolytes included sodium: 140 mEq/l and potassium: 3.7 mEq/l. Troponin was negative. During his admission PCR was sent for COVID-19 which was positive. During the time the patient was diagnosed with COVID-19, dose of mycophenolic acid was decreased from 1440 to 1080 mg daily.

During his admission, the patient was given hydroxychloroquine, oseltamivir and imipenem. The patient did not report any severe symptoms and after three days of hospital stay he was discharged with a good condition.

### Case number 2

A 35 year old male, who was the identical twin brother of case number 1, developed fever, chills, headache, mild dyspnea and an objective loss of sense of smell and taste. The patient developed his symptoms five days prior to his brother.

The patient had no history of previous illnesses and was otherwise healthy. The patient worked in the same office as his brother and mentioned symptoms suspicious of COVID-19 among his coworkers.

The patient referred to a local physician and blood tests and a chest CT scan was requested. He had a normal CBC, oxygen saturation of 91%, and a CRP of 52 mg/l. His chest CT scan was in favor of COVID-19 (Fig. 1).

Considering that the patient did not have any severe symptoms and had a good overall condition he was not admitted to the hospital and was advised to self-quarantine at home with hydroxychloroquine and oseltamivir.

Figure 1 shows a side to side CT scan image of the chest from both brothers (Fig. 1).

## Discussion and conclusions

The Transplantation Society recently acknowledge a gap in data related to COVID-19 in organ transplantation, however advised that patients who are lymphopenic maybe at higher risks of developing severe forms of the disease and considering that patients with transplantations may have degrees of drug-induced lymphopenia, these individuals have to be closely monitored [4]. Our specific patient did not show any severe symptoms and did not show any abnormal lab tests beside his initial CRP which was 32 which became negative during his hospital admission. This maybe supporting the fact that perhaps lymphopenia is a manifestation of the severe form of the disease and not necessarily a predisposing risk factor for the disease itself although stronger evidence is needed to support this claim.

Although information on COVID-19 clinical course among individuals with organ transplantations is largely missing, the comparison of our two cases who were identical twins that contracted COVID-19 showed that the brother who had liver transplantation mentioned having a better overall condition than his twin brother. This may be showing that patients who are receiving immunosuppressive medication may not be at higher risks of developing severe symptoms related to the virus. Whether this phenomena is related to the organ of transplantation (liver) or related to the immunosuppressive medication is yet to be answered.

Although more cases need to be studied, identical twins are a great example for comparison as they have similar physiological characteristics and are expected to respond in a similar manner to COVID-19.

Our patient who had liver transplantation and COVID-19 did not present more severe symptoms compared to his counterpart without liver transplantation and did not need to be hospitalized or be given antiviral drugs for COVID-19.

## Data Availability

N/A.

## Abbreviations

MELD: End-Stage Liver Disease
CRP: C-reactive protein
CT: Computed tomography
WBC: White blood cell count
INR: International normalized ratio
PTT: Partial thromboplastin time
BUN: Blood urea nitrogen
AST: Aspartate transaminase
ALT: Alanine transaminase
ALK: Alkaline phosphatase
